# East‐facing *Helianthus annuus* has maximal number and mass of kernel‐filled seeds: Seed traits versus head orientation

**DOI:** 10.1002/pei3.10083

**Published:** 2022-06-29

**Authors:** Péter Takács, Judit Slíz‐Balogh, Dénes Száz, Gábor Horváth

**Affiliations:** ^1^ Department of Biological Physics ELTE Eötvös Loránd University Budapest Hungary

**Keywords:** absorbed light energy, eastward orientation, *Helianthus annuus*, inflorescence, reproductive fitness, seed number and mass, seed traits, sunflower

## Abstract

After anthesis, the majority of mature sunflower (*Helianthus annuus*) inflorescences face constantly East, which direction ensures maximal light energy absorbed by the inflorescences in regions where afternoons are on average cloudier than mornings. Several theories have tried to explain the function(s) of this eastward orientation. Their common assumption is that eastward facing has certain advantages for sunflowers. In sunflower plantations, the capitulum of many plants can also face North, South, or upward. Large deviations from the conducive East direction can decrease the plant's reproductive fitness. A larger mass and number of seeds, for example, can guarantee safer seed germination and better early development of more offspring. Thus, our hypothesis was that the East facing of sunflower inflorescences ensures a larger seed number and mass compared to disoriented inflorescences. This idea was tested in a sunflower plantation, where we compared the number and mass of seeds in plants, the inflorescences of which were naturally or artificially oriented northward, eastward, southward, westward, or upward. Our study tested head diameter, seed weight, and seed number in a normal agronomic field setting being different from earlier investigations. The other difference was that we tested five head orientations and only East showed significantly increased seed weight and number. Using radiational computations, we showed that East facing ensures more absorbed light energy than other orientations, except upward. This finding can be one of the reasons for the maximal seed number and mass in East‐facing sunflower capitula. Although upward‐facing horizontal inflorescences absorbed maximal light energy, they had the fewest and lightest seeds probably because of the larger temperature and humidity as well as the too much sunlight, all three factors impairing the normal seed development. This study is the first that compares the seed traits of all head orientations of *Helianthus annuus* and proposes that the absorbed radiation could play a major role in the maximal seed number and mass of east‐facing heads.

## Graphical abstract

Mature sunflower (*Helianthus annuus*) inflorescences no longer follow the Sun, and their majority face nearly East. This eastward orientation may have certain advantages for sunflowers. The eastern direction ensures maximal light energy absorbed by the inflorescences, if on average afternoons are cloudier than mornings, as is the usual meteorological situation averaged for decades. We hypothesized that the East facing of sunflower inflorescences ensures larger seed number and mass compared to disoriented inflorescences. In a sunflower plantation we found that East‐facing sunflower heads had much more and heavier seeds than heads facing North, South or West. Upward‐facing horizontal inflorescences had the fewest and lightest seeds, because they might have received too much sunlight which could impair the normal seed development.

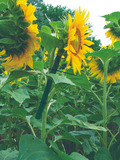

## 1 INTRODUCTION

After anthesis, the mature head of sunflowers (*Helianthus annuus* Linnaeus 1753) no longer follows the Sun's celestial motion, and its inflorescence (capitulum) faces constantly and near‐uniformly East (Atamian et al., 2016; Darwin & Darwin, 1897; Vandenbrink et al., 2014). The natural eastward orientation of the capitula is imposed by complex interactions between the plant's circadian clock and environmental cues (Atamian et al., 2016; Creux et al., 2021). Using drone photography, Takács et al. (2022) showed that the average azimuth direction of the normal vector of mature sunflower inflorescences is almost exactly the geographical East, rather than the azimuth of local sunrise. This geographical eastward direction of mature sunflower heads ensures maximal light energy absorbed by the inflorescences in regions where afternoons are on average cloudier than mornings (Horváth et al., 2020), which is typical for the eastern North American domestication area of sunflowers (Blackman et al., 2011).

The maximum scatter of the azimuth direction of the capitula can even reach ±90° (Takács et al., 2022). The consequence of such large disorientations from the conducive eastward direction is that in a sunflower plantation, the capitulum of many plants can face the geographical North, South, or upward. However, naturally westward‐facing inflorescences are very rear according to our own observations. It is likely that these differently oriented sunflowers are governed by phototropism and would not receive optimal light if they were in the easterly orientation, so they oriented to the directions where most light was available for them. The lack of western head direction can be explained by the phenomenon that this direction does not provide optimal radiation for sunflower development in regions with cloudier afternoons (Horváth et al., 2020). Also, the circadian gating of the stems response to light ensures that almost no west‐facing plants would be naturally present in plantations (Atamian et al., 2016).

Large deviations from the energetically conducive eastern direction may result in a reduction of the plant's reproductive fitness due to the decreased light energy absorbed by the sunflower inflorescence. Recently, Creux et al. (2021) experimentally manipulated sunflower capitulum orientation and temperature under field and controlled conditions. They studied naturally East‐oriented capitula and artificially reoriented West‐facing capitula. They found that East‐facing capitula had faster style elongation, pollen presentation, and pollinator visits. Furthermore, these inflorescences sired more offspring and in one (Davis, CA, USA) of the two study sites (Davis + Charlottesville, VA, USA) produced heavier and plumper seeds compared to capitula forced to orient westward. Mainly the local ambient temperature on the capitulum regulated the timing of style elongation, pollen emergence, and pollinator visits. Note that Charlottesville is cloudier and receives less radiation than Davis, and this supports our hypothesis proposed in this work that cloudier afternoons are linked to lower seed weights and numbers in differently oriented sunflowers.

There are at least eight theories that try to explain the advantages/functions of the eastward orientation of mature sunflower inflorescences: (1) decreased heat stress at noon (Lang & Begg, 1979; Leshem, 1977), (2) reduced chance of fungal attack due to greater reception of sunlight in the early morning that accelerates the drying of morning dew (Lang & Begg, 1979), (3) lower head temperature that could be advantageous for seed maturation and grain filling (Ploschuk & Hall, 1995), (4) decreased heat load in afternoon periods of high irradiance (Seiler, 1997), (5) reduced seed predation by birds (Seiler, 1997), (6) increased attractiveness to pollinators because of increased morning interception of sunlight (Lamprecht et al., 2007), (7) maximum light energy absorbed by east‐facing inflorescences, if afternoons are usually cloudier than mornings (Horváth et al., 2020), and (8) coordination of the timing of pollen emergence and pollinator visits for optimal pollination (Creux et al., 2021).

The common assumption of these theories is that the conducive East facing of mature sunflower capitula has certain advantage(s) for the plant. Larger seed mass *m* can guarantee safer seed germination and better early development of the offspring. Furthermore, a greater seed number *N* ensures more offspring. In addition to fitness values, the seed number and seed mass are also important from the point of oilseed production and yields. Thus, our working hypothesis was that the East facing of a sunflower inflorescence results in maximal *m* and *N* of seeds compared to seeds of disoriented capitula. In order to test this idea, in a sunflower plantation, we measured and compared the seed number *N* and seed mass *m* of plants, the capitula of which were naturally or artificially oriented northward, eastward, southward, westward, or upward.

## 2 MATERIALS AND METHODS

We performed our field experiment from July 1 to August 31, 2021 in a sunflower plantation of the Agricultural Cooperative in Sződ (47° 43′ North, 19° 12′ East, northern Hungary). The plantation was rain‐fed (not irrigated) without chemical soil treatment or fertilization. The planting happened in the second week of April 2021. The density of sunflowers was 9/m^2^, the spacing between rows was 50 cm, the plants were 50 cm apart in a row, and the parallel rows were oriented 25° clockwise from the geographical East. The dimension of the plantation was about 200 m × 700 m. The anthesis began in the first week of July and the maturity of heads happened in the last week of August. The heads were harvested on September 4 for data collection. Although air temperature and precipitation were not registered during the experiment, this could not be a serious problem, because the studied 50 sunflowers (see later) were in an area of 50 m × 50 m, thus they received practically the same temperature and rain.

The hybrid type of the studied sunflowers (*Helianthus annuus*) is Corteva (earlier named Pioneer) P64LE25 with the following characteristics: (1) highly productive, stable, reliable hybrid, (2) early maturity date, (3) resistant against the downy mildew strains and sunflower broomrape, thus it has a Pioneer Protector® Downy Mildew and Broomrape qualification, (4) possesses the feature of ExpressSun®, thus enables safe weed‐killing, (5) on the semi‐floppy tilted head slacking rainwater cannot pile (in which various pathogens could proliferate), (6) information is not available about the cross‐pollination by insects and the degree of self‐pollination.

**FIGURE 1:**
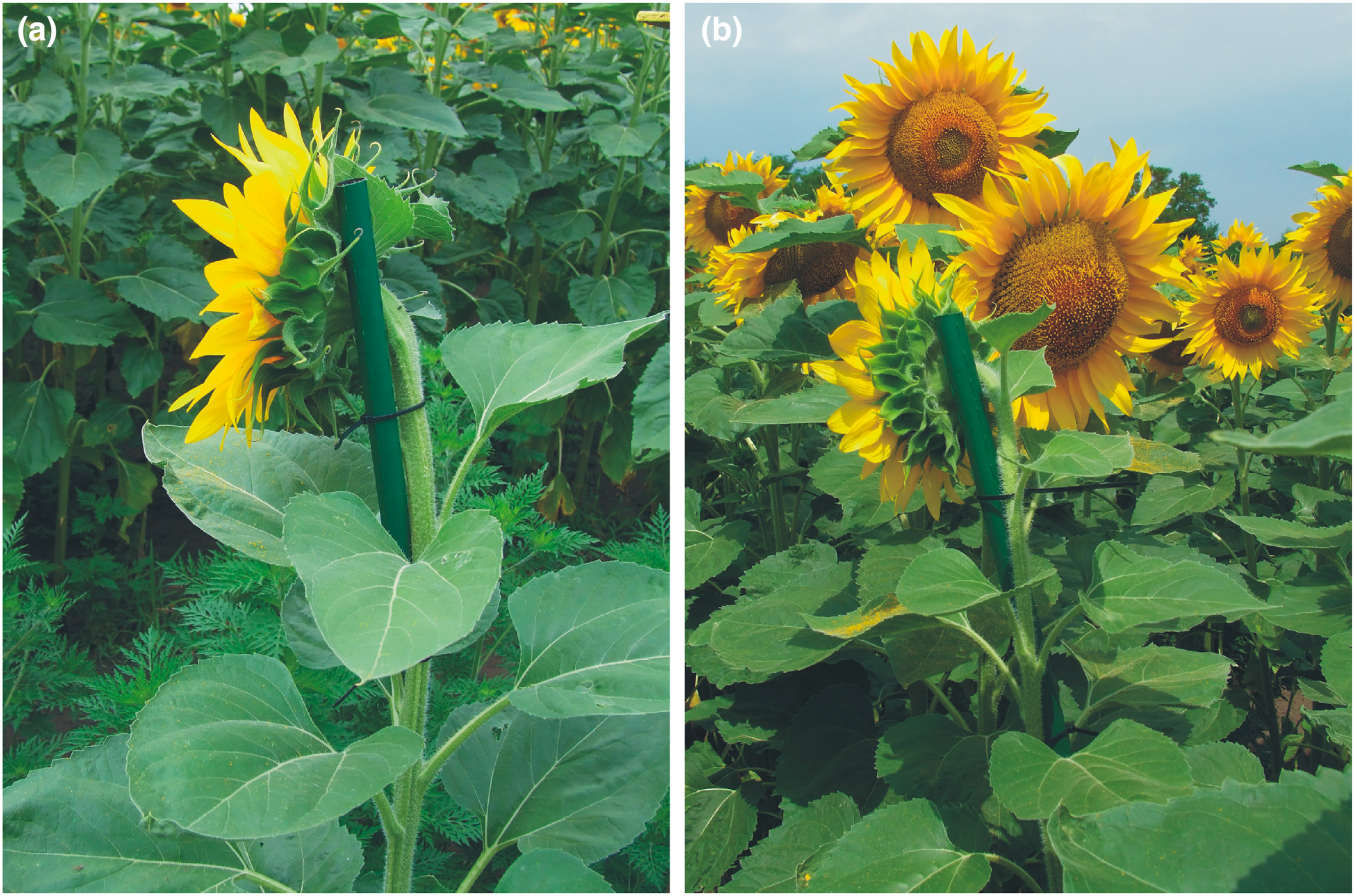
(a, b) Photograph (taken by Gábor Horváth) of a sunflower inflorescence forced to turn westward by a green metal tube (mass = 35 gram, length = 20 cm, diameter = 2 cm) fixed to the stem by two black plastic bonds among naturally east‐facing sunflowers

The sunflowers in the above‐mentioned plantation were monitored for the cessation of heliotropism just before the onset of anthesis (first week of July), and at this time, we selected a row in the plantation's center, where the developmental condition of the plants was the same. Using tubes, in this row, we forced 10 immature (still nonflowering) heads to turn West, named tW (i.e., tube‐wearing and West‐facing) further on. The artificial twist of a given head was attained with a light metal tube (mass = 35 g, length = 20 cm, and diameter = 2 cm) fixed by two plastic bonds to the stem in such a way that they do not hinder the normal development of the plant (Figure 1). In order to hinder that the plastic bonds can cut into the expanding stem, we have periodically slightly loosen the bonds in such a way that the head cannot turn from its desired/prescribed direction. At the end of the breeding season, on September 4, 2021, we harvested the 10 artificially West‐facing tube‐wearing tW heads for laboratory investigation of their seeds.

In the studied sunflower plantation, we observed that several sunflower heads had irregular orientations differing considerably from the typical eastward direction. The inflorescences of some of these irregular heads faced exactly North, South, or upward, but naturally West‐facing heads did not occur. This was the reason why we forced 10 sunflower inflorescences (tW) to turn artificially westward by tubes. On September 4, 2021, we also harvested 10 naturally North‐facing (nN), 10 naturally South‐facing (nS), 10 naturally East‐facing (nE), and 10 naturally Upward‐facing (nU) heads for laboratory measurements from an area of 50 m × 50 m containing the 10 tW sunflowers. Since within this area, there were no larger visible anatomical differences (e.g., in plant height, leaf size, color) among plants, it is improbable that the reason for our results (see later) could be the spatially different soil fertility or soil moisture, for example. Hence, after harvest, we studied the ripe seeds of the following five types of sunflower heads:

- nN: naturally North‐facing
- nE: naturally East‐facing
- nS: naturally South‐facing
- nU: naturally Upward‐facing
- tW: tube‐wearing, artificially West‐facing

We used the tube treatment only at the artificially West‐facing sunflower heads because there were no heads that faced naturally West, thus we had to twist toward West the 10 selected heads by tubes. On the other hand, we wanted to minimize the number of tube‐manipulated sunflowers, since this tube treatment might have slightly hindered the development of plants.

**FIGURE 2:**
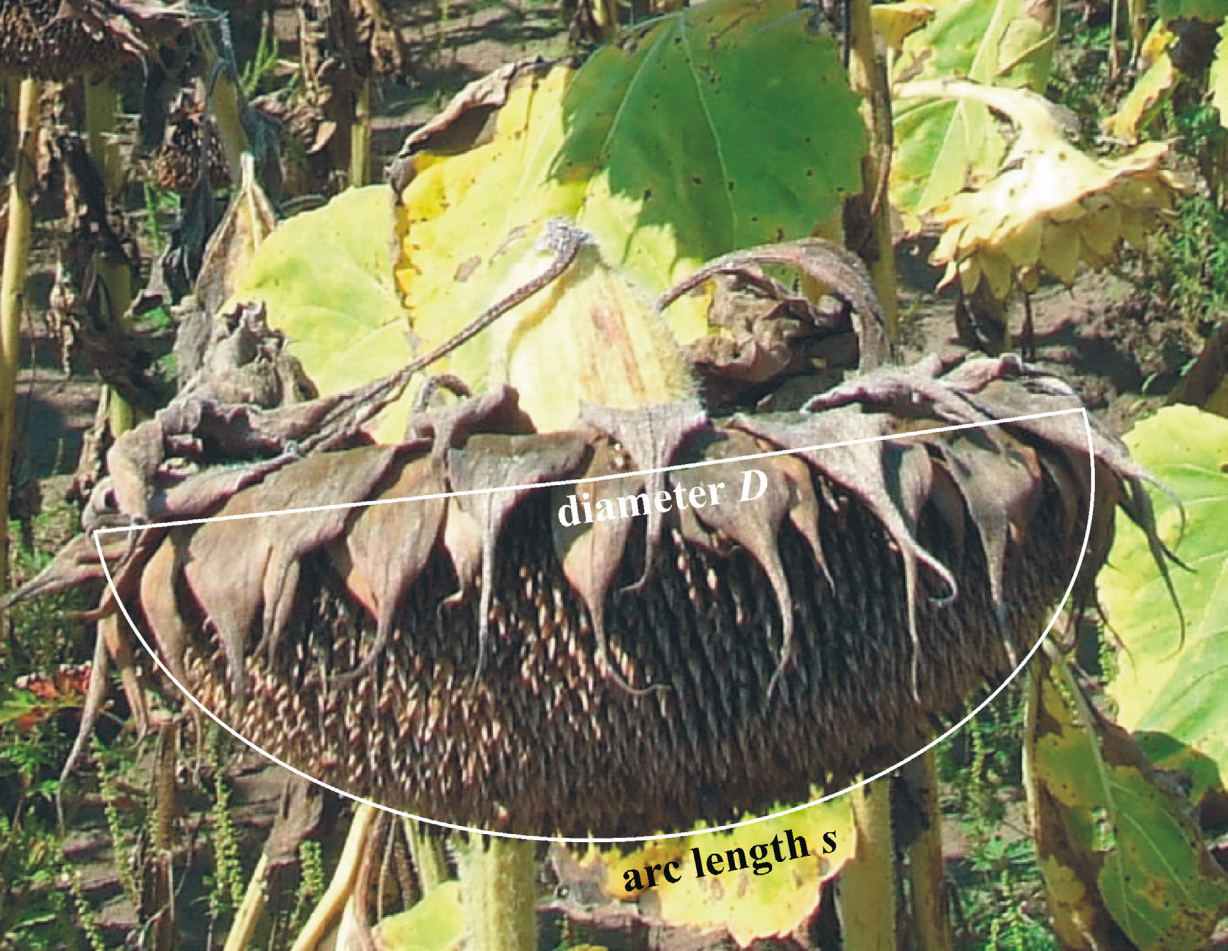
Definition of the diameter *D* and arc length *s* along the diameter of a mature sunflower head (photographed by Gábor Horváth)

The diameter *D* and arc length *s* along the diameter of the collected 5 × 10 sunflower heads (Figure 2) were measured with a flexible measuring tape in the laboratory. The seeds filled with kernel (= dehulled seed) and unfilled seeds (without kernel, found mainly in the outermost circular row of the head) of the collected 50 sunflower heads were separated from each head and they were counted, resulting in the number *N*_type,i_ of kernel‐filled seeds and *U*_type,i_ of unfilled seeds, where type = nN, nE, nS, nU, tW; i = 1, 2, …, 10 in the *i*th head of a given type. The mass of unfilled seeds was not measured because of their negligible number relative to the number of kernel‐filled seeds. The seeds were dried for 30 days at a constant room temperature of 20°C in the laboratory. After drying the seeds, the total mass *M*_type,i_ of all (*N*_type,i_) kernel‐filled seeds in the *i*th head of a given type were measured by a common digital scale with a nominal precision of ±0.5 g. The individual mass *m* = *M*/*N* of kernel‐filled seeds was also determined. Finally, the average ± standard deviation of *D*, *s*, *N*, *U*, *M*, and *m* in the 10 sunflower heads of a given type was calculated.

We did not measure the kernel width of filled seeds harvested from the North‐, East‐, South‐, West‐, and Upward‐facing sunflower heads, because in a preliminary measurement we experienced that there is a strong positive correlation among the (i) mass *m*_seed_ of the whole seed (kernel with hull), (ii) kernel mass *m*_kernel_, and (iii) kernel width *w*_kernel_: the larger the *m*_seed_, the larger are *m*_kernel_ and *w*_kernel_. Creux et al. (2021) measured the kernel width and mass of 100 dehulled seeds harvested from East‐ and West‐facing capitula. However, knowing the mentioned positive correlation, we considered more important to measure the average and standard deviation of *m*_seed_ of all (ranging between 108 and 2627, depending on head orientation, Tables S1–S5) harvested seeds, rather than only 100 seeds selected as a probe for each treatment.

Although, in our experiment, the sunflower heads were not covered by a net to limit bird damage, this caused no problem, because in the 50 harvested heads we observed no seed loss due to bird predation. According to our field experience, the slot of a seed picked out by a bird from a head can clearly be seen by the naked eye (see figure 6a‐c of Horváth et al., 2020). Consequently, bird predation did not affect our seed number counts.

Contrary to an experiment like that of Creux et al. (2021), in the plantation studied by us, the insect pollination on the 50 selected sunflower inflorescences could not have been controlled. However, as mentioned above, these plants stood in a limited area (50 m × 50 m) in the middle of the plantation, thus, on average, they had practically the same chance to be visited by pollinators, independently of their azimuth direction. So we do not think that the observed differences in seed traits could be explained by pollination effects.

For statistical analysis, we used one‐way ANOVA to test whether the analyzed sunflower groups were different from each other, then we performed a post‐hoc test according to the Tukey–Kramer method to specify the sunflower group‐pairs being significantly different. With this method, we compared the number *N*, the average seed mass *m*, the total mass *M* of kernel‐filled seeds, and the number *U* of unfilled seeds in sunflower heads as well as the average head diameter *D* and diameter's arc length *s* of different types. For statistical computations, we used the statistical function package of Microsoft Excel 2021.

Using the radiation software developed by Horváth et al. (2020), we computed the total light energy *e* per unit area absorbed by a sunflower inflorescence, the normal vector of which is oriented constantly toward the geographical East (with azimuth angle α_East_ = +90° measured clockwise from North), South (α_South_ = +180°), West (α_West_ = −90°), North (α_North_ = 0°), or upward between anthesis (July 1) and senescence (September 7) under the typical cloud conditions of Hungary, where mornings are usually less cloudy than afternoons, just like in the eastern North American domestication region of *Helianthus annuus* (Blackman et al., 2011). This software uses the regional meteorological data of daytime cloudiness between anthesis and senescence, the local astronomical data of the Sun's daily motion in the sky, and the time‐dependent decreasing elevation angle of the normal vector of mature sunflower inflorescences and their absorption spectra. Seeking a radiation explanation of the eastward facing of mature sunflower inflorescences, Horváth et al. (2020) computed only the light energy absorbed by an inflorescence as a function of its azimuth direction, rather than the light energy received by the leaves oriented to all possible azimuths (N, NE, E, ES, S, SW, W, and NW).

## 3 RESULTS

**FIGURE 3:**
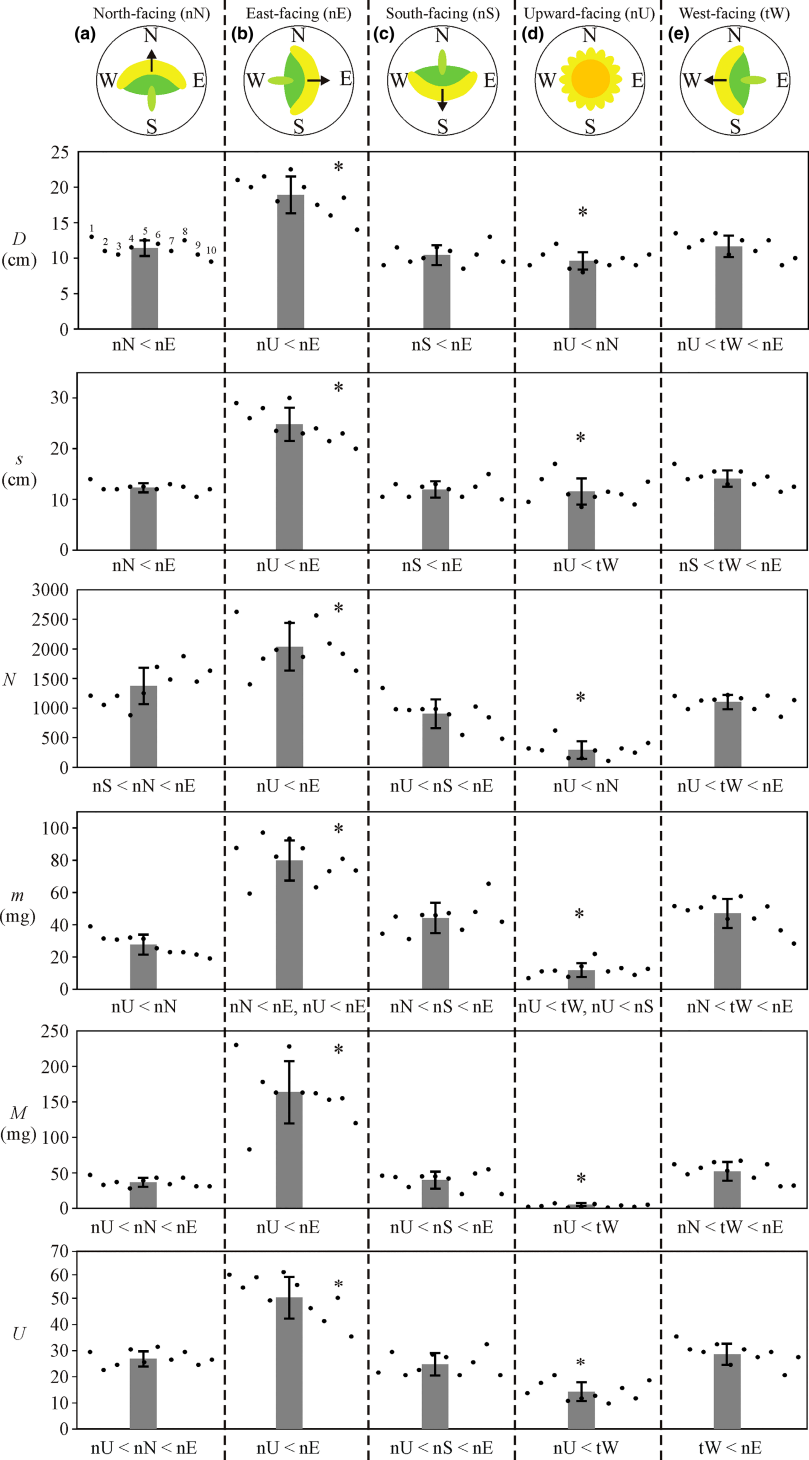
Average (gray bars) ± standard deviation (I‐s) of the measured values (dots) of the head diameter *D* (cm), arc length *s* (cm) along head diameter, number *N*, mass *m* (mg), and total mass *M* = *N·m* (g) of kernel‐filled seeds in 10 sunflower heads of different types: (a) naturally north‐facing (nN), (b) naturally east‐facing (nE), (c) naturally south‐facing (nS), (d) naturally upward‐facing (nU), and (e) tube‐wearing, artificially west‐facing (tW). *U* is the number of unfilled seeds (without kernel). In a given row, the asterisk (*) indicates those averages which are statistically significantly different from all other averages. The relation symbol < indicates statistically significant differences between the values of a given variable for pairwise comparisons (Table 1; Supplementary Statistical Table Groups T1–T3)

According to Figure 3 and Table 1 (see also Tables S1–S5, Supplementary Statistical Table Groups T1‐T3), the naturally East‐facing nE heads had maximal average diameter *D*, arc length *s* along the diameter, number *N*, mass *m*, and total mass *M* = *Nm* of kernel‐filled seeds, and maximal average number *U* of unfilled seeds, whose maximal values differed significantly from the corresponding smaller values of nN, nS, and nU heads. Minimal average values of *D*, *s*, *N*, *U*, *m*, and *M* had the nU heads, and their minimal *N*‐, *U*‐, *m*‐, and *M*‐values differed significantly from the larger corresponding values of heads nN, nS, nE, and tW. *D* of nU heads was significantly smaller than *D* of nE, tW, and nN heads, furthermore, *s* of nU capitula was significantly smaller than *s* of nE and tW capitula (Table 1; Supplementary Statistical Table Groups T1‐T2). The nN, nS, and tW heads had intermediate *D*‐, *s*‐, *N*‐, *U*‐, *m*‐, and *M*‐values between those of nE and nU heads (Figure 3 and Table 1); Supplementary Statistical Table Groups T1‐T3 contain all possible pairwise comparisons between them obtained from the Tukey–Kramer post‐hoc tests. Both the average *D*‐ and *s*‐values of nE sunflowers were significantly larger than those of nN sunflowers (Supplementary Statistical Table Group T1). While *s* of tW heads was significantly larger than *s* of nS heads, there was no significant difference in *D* between tW and nS heads. Furthermore, *D* of nN plants was significantly larger than *D* of nU plants, and there was no significant difference in *s* between nN and nU plants.

**TABLE 1:** Average ± standard deviation (designed by Δ) of the head diameter *D* ± Δ*D* (cm), arc length *s* ± Δ*s* (cm) along head diameter, number *N* ± Δ*N*, mass *m* ± Δ*m* (mg), and total mass *M* ± Δ*M* (g) of kernel‐filled seeds in sunflower heads of types nN (naturally north‐facing), nE (naturally east‐facing), nS (naturally south‐facing), nU (naturally upward‐facing), and tW (tube‐wearing, artificially west‐facing)

Variable	Sunflower head type				
	nN	nE	nS	nU	tW
*D* (cm)	11.4 (60.3%) nN < nE	18.9* (100%) nU < nE	10.4 (55.0%) nS < nE	9.6* (50.8%) nU < nN	11.7 (61.9%) nU < tW < nE
Δ*D* (cm)	±1.1 (±5.8%)	±2.6 (±13.8%)	±1.4 (±7.4%)	±1.2 (±6.3%)	±1.5 (±7.9%)
*s* (cm)	12.3 (49.6%) nN < nE	24.8* (100%) nU < nE	12.0 (48.4%) nS < nE	11.6* (46.8%) nU < tW	14.1 (56.9%) nS < tW < nE
Δ*s* (cm)	±0.9 (±3.6%)	±3.3 (±13.3%)	±1.6 (±6.5%)	±2.6 (±10.5%)	±1.6 (±6.5%)
*N*	1373.9 (67.5%) nS < nN < nE	2036.6* (100%) nU < nE	904.7 (44.4%) nU < nS < nE	290.6* (14.3%) nU < nN	1102.7 (54.1%) nU < tW < nE
Δ*N*	±308.8 (±15.2%)	±401.4 (±19.7%)	±244.2 (±12.0%)	±148.3 (±7.3%)	±122.0 (±6.0%)
*m* (mg)	27.7 (34.7%) nU < nN	79.8* (100%) nN < nE, nU < nE	44.2 (55.4%) nN < nS < nE	11.9* (14.9%) nU < tW, nU < nS	47.0 (58.9%) nN < tW < nE
Δ*m* (mg)	±6.2 (±7.8%)	±12.4 (±15.5%)	±9.4 (±11.8%)	±4.2 (±5.3%)	±9.1 (±11.4%)
*M* (g)	36.8 (22.5%) nU < nN < nE	163.7* (100%) nU < nE	39.8 (24.3%) nU < nS < nE	3.5* (2.1%) nU < tW	52.2 (31.9%) nN < tW < nE
Δ*M* (g)	±6.3 (±3.8%)	±21.2 (±13.0%)	±12.1 (±7.4%)	±2.1 (±1.3%)	±13.2 (±8.1%)
*U*	27.6 (53.4%) nU < nN < nE	51.7* (100%) nU < nE	25.4 (49.1%) nU < nS < nE	14.6* (28.2%) nU < tW	29.3 (56.7%) tW < nE
Δ*U*	±3.0 (±5.8%)	±8.2 (±15.8%)	±4.4 (±8.5%)	±3.7 (±7.2%)	±4.2 (±8.1%)

*Note*: *U* ± Δ*U* is the number ± standard deviation of unfilled seeds (without kernel). Further details are available in Tables S1‐S5. Parentheses: The maximal value in a given row is 100%, and the other percentages are calculated relative to this maximum. In a given row, asterisk (*) indicates those averages which are statistically significantly different from all other averages. The relation symbol < indicates statistically significant differences between the values of a given variable for pairwise comparisons (Supplementary Statistical Table Groups T1–T3). In the row of *D*, nN < nE means that *D*_nN_ is significantly smaller than *D*_nE_, for example.

According to Table 2, there was a strong positive correlation between seed number *N* and head diameter *D* as well as between *N* and the diameter's arc length *s* in sunflower heads. This means that wider heads (with larger *D* and *s*) contained more kernel‐filled seeds (greater *N*).

**TABLE 2:** Pearson's correlation coefficient *R* computed between the average number *N* of kernel‐filled seeds and head diameter *D* as well as between *N* and the average diameter's arc length *s* in sunflower heads

*R* (*N* vs *D*)	*R* (*N* vs *s*)	Correlation type
0.74	0.72	Strong positive

According to Table 3, between anthesis (July 1) and senescence (September 7) under the cloud conditions of Hungary (see Figure 2 of Horváth et al., 2020), constantly upward‐facing, horizontal mature sunflower inflorescences absorb much more total light energy *e* per unit area (1089 MJ/m^2^) than tilted inflorescences oriented permanently toward East (155 MJ/m^2^), South (99 MJ/m^2^), West (145 MJ/m^2^), or North (96 MJ/m^2^). Among the four mentioned orientation types, East‐facing inflorescences absorb the largest *e*, and West‐facing ones absorb slightly less light energy. The South‐ and North‐facing inflorescences absorb much less *e* than East‐ or West‐facing ones. Depending on the cloudiness data and radiation (Horváth et al., 2020), the total light energy *e* absorbed by South‐facing inflorescences is practically the same or larger than that absorbed by North‐facing inflorescences (Table 3).

**TABLE 3:** Total light energy *e* per unit area (in MJ/m^2^) absorbed by a mature sunflower inflorescence, the normal vector of which is oriented constantly toward the geographical east (with azimuth angle α_East_ = +90° measured clockwise from north), south (α_South_ = +180°), west (α_West_ = −90°), north (α_North_ = 0°), and upward (to zenith) between anthesis (1 July) and senescence (September 7) under the typical cloud conditions of Hungary computed with the use of the software of Horváth et al. (2020)

Orientation, azimuth α				
East α_East_ = +90°	South α_South_ = +180°	West α_West_ = −90°	North α_North_ = 0°	Upward (to zenith)
154.5 MJ/m^2^	98.7 MJ/m^2^	144.5 MJ/m^2^	98.9 MJ/m^2^	1088.5 MJ/m^2^

Both South‐facing (nS) and North‐facing (nN) inflorescences absorbed *e* ≈ 100 MJ/m^2^ light energy, while East‐facing (nE) and West‐facing (tW) ones received nearly 50% more light (*e*_nE_ ≈ 155 MJ/m^2^ > *e*_tW_ ≈ 145 MJ/m^2^). From this, based on our hypothesis that the amount of absorbed radiation significantly affects the mass *m* of kernel‐filled seeds, one can expect that (i) nE and tW capitula produce higher *m* compared to nS and nN capitula, and (ii) nE heads develop higher *m* than tW ones. According to Table 1 and Figure 3, both expectations are realized in our experiment, because the following significant differences are true for pairwise comparisons of *m*: *m*_nN_ < *m*_nE_, *m*_nN_ < *m*_nS_ < *m*_nE_, *m*_nN_ < *m*_tW_ < *m*_nE_. Very similar relations are true for the number *N* of kernel‐filled seeds (*N*_nS_ < *N*_nN_ < *N*_nE_, *N*_tW_ < *N*_nE_), head diameter *D* (*D*_nN_ < *D*_nE_, *D*_nS_ < *D*_nE_, *D*_tW_ < *D*_nE_), and arc length *s* along diameter (*s*_nN_ < *s*_nE_, *s*_nS_ < *s*_tW_ < *s*_nE_). All these significant results support our proposition that sunflower traits *m*, *N*, *D*, and *s* (strongly positively correlated with each other, Table 2) are more likely regulated by radiation exposure.

Although upward‐facing nU capitula absorbed the most light energy *e*_nU_ ≈ 1089 MJ/m^2^ (Table 3), they produced the smallest *m* (*m*_nU_ < *m*_nN_, *m*_nU_ < *m*_nE_, *m*_nU_ < *m*_tW_, *m*_nU_ < *m*_nS_), *N* (*N*_nU_ < *N*_nE_, *N*_nU_ < *N*_tW_, *N*_nU_ < *N*_nN_, *N*_nU_ < *N*_nS_), *D* (*D*_nU_ < *D*_nN_, *D*_nU_ < *D*_tW_, *D*_nU_ < *D*_nE_), *s* (*s*_nU_ < *s*_tW_, *s*_nU_ < *s*_nE_) of kernel‐filled seeds, and *U* (*N*_nU_ < *N*_nE_, *N*_nU_ < *N*_tW_, *N*_nU_ < *N*_nN_, *N*_nU_ < *N*_nS_) of unfilled seeds compared to capitula with other orientations (Table 1, Figure 3). One of the reasons for this could be that nU inflorescences received too much radiation. Since the highest and lowest thresholds in radiation required for normal sunflower seed development are not known, the study of these thresholds is an interesting task of future research.

## 4 DISCUSSION

Most of the earlier investigations have speculated on or studied experimentally some advantageous effects of the East facing of sunflower inflorescences on the capitulum microclimate (Lang & Begg, 1979; Leshem, 1977; Ploschuk & Hall, 1995; Seiler, 1997), the protection against fungal attack (Lang & Begg, 1979), and avian seed predation (Seiler, 1997) or pollinator visits (Atamian et al., 2016; Creux et al., 2021; Lamprecht et al., 2007). It has also been suggested that cloud cover, strongly influencing the total amount of solar radiation received, could impact sunflower seed development (Rawson et al., 1984). Unlike these previous studies, we conducted an end‐point analysis of two of the most important seed traits, the seed number, and mass of 5 × 10 capitula in five different directions (North, East, South, West, and upward) in a sunflower plantation developing under natural conditions. Earlier, Creux et al. (2021) measured seed weight and number for 10 plants each for East and West across two different trials which means a total of 20 plants were assayed per treatment in each location. They found no difference in the seed number *N* produced by East‐facing or West‐facing sunflower capitula. They concluded that *N* is affected by location and planting time in the season but not by final capitulum orientation.

Our experiment was performed in a field plantation similar to commercial production settings, which may be distinct from other previous studies. Our study demonstrated that East‐facing sunflower heads perform superior to North‐, South‐, West‐, and upward‐facing ones. We found that naturally East‐facing nE sunflower heads had significantly more kernel‐filled seeds than naturally North‐, South‐, upward‐facing nN, nS, nU heads or artificially West‐facing tW heads (Figure 3 and Table 1; Supplementary Statistical Table Group T2). We suggest that naturally East‐facing nE heads could develop more seeds than nN, nS, and tW heads due to the fact that nE inflorescences absorbed more total light energy than nN, nS, and tW inflorescences (Table 3).

Note that our field study was performed in a single location (in Hungary) and single season (in 2021) in sunflowers grown directly on the soil in a plantation in a standard agricultural setting following agronomic practices such as high‐density and row‐cropping, for example. On the other hand, Creux et al. (2021) conducted a two‐location and two‐season study by sunflowers grown in isolated and widely spaced large pots or paint buckets. These differences could lead to differences in seed traits such as seed number, as the plants directly in the ground can grow a larger root system and have more access to nutrients or water. As these two experiments happened under two different growing conditions and/or microenvironments, a direct comparison between them cannot be done. Another likely difference between these studies may be the genetics of sunflower cultivars used in these studies. Commercial cultivars are usually improved for aggregate seed yield involving both seed number and weight.

In their Davis trials, Creux et al. (2021) observed that the East‐facing capitula were on average 2 cm larger in diameter *D* than their West‐facing counterparts. The difference in *D* was primarily due to seed size rather than seed number, as kernels harvested from East‐facing capitula were each 0.5 mm wider on average than kernels harvested from West‐facing capitula. We also found that naturally East‐facing sunflower heads nE had significantly larger diameter *D* and diameter's arc length *s* than heads with other natural (nN, nS, and nU) or artificial (tW) orientations (Figure 3 and Table 1; Supplementary Statistical Table Group T1). These differences in *D* and *s* were primarily due to the seed number *N*, because heads with larger *D* and *s* contained more seeds (Table 2). Due to the different growth conditions between the study of Creux et al. (2021) and our field experiment, it is imaginable that the increased head diameter observed by us is a combination of seed number and width, because plants grown in the soil could be larger and more robust than the drip‐irrigated pot‐grown plants investigated by Creux et al. (2021).

In Davis, Creux et al. (2021) found that seeds from East‐facing capitula were on average 20% heavier than those from West‐facing capitula. By contrast, they observed no significant differences in kernel weight or kernel width between kernels harvested from East‐facing and West‐facing capitula grown in Charlottesville. Therefore, they concluded that capitulum orientation has location‐specific, environment‐dependent effects on seed weight. The radiation data provided in Figure S1 of Creux et al. (2021) for Charlottesville and Davis supports our idea that radiation plays an important role in seed development and number.

We also found that naturally East‐facing sunflower heads nE had significantly larger mass *m* of kernel‐filled seeds than heads with other natural (nN, nS, and nU) or artificial (tW) orientations (Figure 3 and Table 1; Supplementary Statistical Table Group T2). We suggest that naturally East‐facing nE heads could develop heavier seeds than nN, nS, and tW heads, because the nE inflorescences absorbed more total light energy than nN, nS, and tW inflorescences (Table 3). Seed development is also significantly affected by the photosynthesis in the leaves producing the sugars required for seeds (e.g., Rawson et al., 1984). Since mature leaves do not follow the sun and orient practically in all possible azimuth directions (from North through East and South to West), the only relevant difference among the nE, nN, nS, and tW heads studied by us was their azimuth orientation. Thus, the observed differences between the seed weights of our differently oriented heads can mainly be explained by our suggestion that seed development is dependent on radiation absorption of the inflorescence. It is an important future task to explore the biology of how radiation might influence seed weight, and whether the role of temperature or light is the more important.

The inflorescences of naturally upward‐facing horizontal nU heads absorbed 7–14.5 times more light energy *e* per unit area than the nN, nE, nS, and tW inflorescences (Table 3). Such a huge amount of light might have been too much which impaired the inflorescence and normal seed development. It is well known that high levels of light and temperature can negatively impact the flowering processes, pollination, and seed development of sunflowers and other flowering plants (e.g., Creux et al., 2021; Ploschuk & Hall, 1995; Rawson et al., 1984). In our opinion, this can also explain our finding that the nU heads had the fewest (least *N*) and lightest (smallest *m*) seeds (Figure 3, Table 1). Alternatively, the upward facing of sunflower heads could increase the temperature or desiccation of inflorescences, which may be disadvantageous for the normal development of reproductive organs and/or embryos. The nU microenvironment may not also be favorable to insect pollinator visitation either due to a reduction in rewards (like nectar volume), or atypical warmer heads for foraging, or odd spectral cues (like ultraviolet floral guides), or achromatic cues. Thus, the absorbed light energy could be only one of the potential causes of the poor performance in nU orientations.

In our experiment, we used tubing to force 10 capitula to face West, by which we might have confounded the West‐facing effect with the effect of tube manipulation. Since there were numerous sunflowers, the inflorescences of which faced naturally East, South, North, or upward, we did not include plants that also had tubes forcing capitula to stay facing these directions. Thus, the results obtained for the tube‐wearing West‐facing capitula (tW) may contain a tube effect. The most probable effect of tube treatment is the irregular head development resulting in significantly reduced head diameter *D*, diameter's arc length *s*, seed numbers *N* and *U*, and seed masses *m* and *M*. However, according to the statistical analyses (Table 1; Supplementary Statistical Table Groups T1‐T3), there were no such unambiguous significant reductions in West‐facing heads (tW) compared to North‐, South‐, and upward‐facing ones (nN, nS, and nU): (i) *D* and *N* were significantly larger in tW than in nU; *s* was significantly larger in tW than in nU and nS; *m* and *M* were significantly larger in tW than in nU and nN. (ii) There were no significant differences in *D*, *N*, and *U* between tW, nN, nS, as well as in *s*, *m*, and *M* between tW and nS. (iii) nE had significantly larger *D*, *s*, *N*, *U*, *m*, and *M* than tW. Consequently, the chance of tube effects resulting in the decrease of head and seed traits in tW sunflowers was highly improbable.

Let us set our findings in a broader context, concerning some possible advantages of the East facing mature sunflower inflorescences: (1) The decreased heat stress of East‐facing sunflower inflorescences at noon assumed by Leshem (1977) and Lang and Begg (1979) could result in better development of seeds. (2) The hypothetic reduced chance of fungal attack of East‐facing sunflower capitula due to greater reception of sunlight in the early morning suggested by Lang and Begg (1979) may accelerate the drying of morning dew, which would obviously be advantageous for seed traits. (3) According to Ploschuk and Hall (1995), the possibly lower head temperature of the East‐facing capitula could be advantageous for seed maturation and grain filling. (4) The maybe decreased heat load of East‐facing inflorescences in afternoon periods of high irradiance surmised by Seiler (1997) could be advantageous for the development of seeds. (5) The alleged reduced seed predation by birds due to East facing of sunflower capitula hypothesized by Seiler (1997) has nothing to do with the seed traits. (6) The perhaps enhanced attractiveness of East‐facing inflorescences to pollinators due to increased morning interception of sunlight proposed by Lamprecht et al. (2007) surely increases the pollination success of florets and thus enhances the seed number. (7) Horváth et al. (2020) showed that the cloud cover strongly influences the total amount of solar radiation received by sunflower inflorescences, and compared to other points of the compass, East‐facing capitula absorb maximum light energy, if on average afternoons are cloudier than mornings. In this work, we demonstrated that this light‐energetical phenomenon impacts the seed development—as Rawson et al. (1984) suggested—in the form of maximal seed number and mass of East‐facing sunflower heads.

There might have been an impact of pollinators on the seed numbers of the differently oriented sunflower capitula studied by us: Differently oriented inflorescences are differently illuminated by direct sunlight, therefore, they have different appearances to pollinators, partly because of the UV markings of the bright yellow petals. This has a major effect on the attractiveness of the flower to insect pollinators. Since pollinators can increase the seed set in sunflower plantations, this might have affected the different seed numbers observed by us. Instead of speculating about the possible consequences of this effect, we mention only that on every day of our field experiment pollinators had the chance to visit the sunflower inflorescences in any time, from morning through noon to afternoon. Thus, during the day, an inflorescence with a given orientation was confronted with the same or similar illumination conditions as another one with another orientation. Consequently, on a given day, differently oriented inflorescences had the same or similar series of visual appearances and attractiveness to pollinators. The only relevant difference is the temporal subalternation of appearances. For example, a sunlit East‐facing inflorescence has the same appearance at 3 h before noon as a sunlit West‐facing inflorescence 3 h after noon. On the other hand, North‐facing inflorescences are sunlit for shorter periods than East‐, South‐, or West‐facing ones, while upward‐facing inflorescences are illuminated by direct sunlight for the longest periods.

## 5 CONCLUSIONS

On the basis of our results we conclude the following:

- Naturally East‐facing (nE) sunflower heads had significantly larger average diameter *D*, diameter's arc length *s*, number *N*, mass *m*, and total mass *M* of kernel‐filled seeds than naturally North‐facing (nN), South‐facing (nS), upward‐facing (nU), or artificially West‐facing (tW) heads.
- nU heads had significantly smaller *N*, *U*, *m*, and *M* of seeds than nN, nE, nS, and tW heads.
- Larger heads contained more filled seeds.
- Under the cloud conditions of Hungary, the total light energy *e* absorbed by mature sunflower inflorescences facing East (E), West (W), South (S), North (N), or upward (U) between anthesis and senescence decreases in the following order: *e*_U_ > *e*_E_ > *e*_W_ > *e*_S_ = *e*_N_.
- nE heads might have developed more and heavier seeds than nN, nS, and tW heads, because the former absorbed more total light energy than the latter.
- Horizontal nU heads had the fewest and lightest seeds, because the 7–15 times more light energy relative to nN, nE, nS, and tW heads impaired the normal seed development.

## CONFLICT OF INTEREST

The authors have no conflict of interest.

## Supporting information


**Appendix S1.** Supporting information.

## Data Availability

The data that supports the findings of this study are available in the supplementary material of this article.
