# Supplementary material for: East‐facing Helianthus annuus has maximal number and mass of kernel‐filled seeds: Seed traits versus head orientation
Source: Plant Environ Interact. 2022 Jun 29;3(3):130–9. doi: 10.1002/pei3.10083 (PMC10168033; doi:10.1002/pei3.10083)
Supplement: Supplementary file 1 — Appendix S1. Supporting information. [file PEI3-3-130-s001.docx]

**Supporting Information**

for

**East-facing *Helianthus annuus* has maximal number and mass of kernel-filled seeds: seed traits versus head orientation**

Péter Takács, Judit Slíz-Balogh, Dénes Száz and Gábor Horváth^1,*^

1: Department of Biological Physics,

ELtE Eötvös Loránd University, H-1117 Budapest, Pázmány sétány 1, Hungary

^*^Corresponding author: gh@arago.elte.hu

This file contains the following: - Supplementary Tables S1, S2, S3, S4, S5

- Supplementary Statistical Table Groups T1, T2, T3

**Supplementary Table S1**: Number *N* and total mass *M* of all kernel-filled seeds, furthermore diameter *D* and arc length *s* along the diameter in 10 sunflower heads (i = 1, 2, ..., 10) of type nN = naturally North-facing. *U* is the number of unfilled seeds (without kernel).

**sunflower head type nN = naturally North-facing**

| **head** | ***N*_i_** | ***M*_i_ (g)** | ***m*_i_ = *M*_i_/*N*_i_ (mg)** | ***D*_i_ (cm)** | ***s*_i_ (cm)** | ***U*_i_** |
| --- | --- | --- | --- | --- | --- | --- |
| **i = 1** | 1209 | 47.2 | 39.0 | 13 | 14 | 30 |
| **i = 2** | 1054 | 33.2 | 31.5 | 11 | 12 | 23 |
| **i = 3** | 1207 | 37.2 | 30.8 | 10.5 | 12 | 25 |
| **i = 4** | 880 | 28.2 | 32.0 | 11.5 | 12.5 | 31 |
| **i = 5** | 1252 | 39.2 | 31.3 | 12.5 | 12.5 | 26 |
| **i = 6** | 1696 | 43.2 | 25.5 | 12 | 12 | 32 |
| **i = 7** | 1483 | 34.2 | 23.1 | 11 | 13 | 27 |
| **i = 8** | 1878 | 43.2 | 23.0 | 12.5 | 12.5 | 30 |
| **i = 9** | 1448 | 31.2 | 21.5 | 10.5 | 10.5 | 25 |
| **i = 10** | 1632 | 31.2 | 19.1 | 9.5 | 12 | 27 |
| **average** | ***N*** | ***M* (g)** | ***m* (mg)** | ***D* (cm)** | ***s* (cm)** | ***U*** |
|  | 1373.9 | 36.8 | 27.7 | 11.4 | 12.3 | 27.6 |
| **standard**  **deviation** | **Δ*N*** | **Δ*M* (g)** | **Δ*m* (mg)** | **Δ*D* (cm)** | **Δ*s* (cm)** | **Δ*U*** |
|  | 308.8 | 6.3 | 6.2 | 1.1 | 0.9 | 3.0 |

**Supplementary Table S2**: Number *N* and total mass *M* of all kernel-filled seeds, furthermore diameter *D* and arc length *s* along the diameter in 10 sunflower heads (i = 1, 2, ..., 10) of type nE = naturally East-facing. *U* is the number of unfilled seeds (without kernel).

**sunflower head type nE = naturally East-facing**

| **head** | ***N*_i_** | ***M*_i_ (g)** | ***m*_i_ = *M*_i_/*N*_i_ (mg)** | ***D*_i_ (cm)** | ***s*_i_ (cm)** | ***U*_i_** |
| --- | --- | --- | --- | --- | --- | --- |
| **i = 1** | 2627 | 230.2 | 87.6 | 21 | 29 | 60 |
| **i = 2** | 1402 | 83.2 | 59.3 | 20 | 26 | 55 |
| **i = 3** | 1835 | 178.2 | 97.1 | 21.5 | 28 | 59 |
| **i = 4** | 1985 | 163.2 | 82.2 | 18 | 23.5 | 50 |
| **i = 5** | 2443 | 228.2 | 93.4 | 22.5 | 30 | 61 |
| **i = 6** | 1866 | 163.2 | 87.5 | 20 | 23 | 56 |
| **i = 7** | 2566 | 162.2 | 63.2 | 17.5 | 24 | 47 |
| **i = 8** | 2092 | 153.2 | 73.2 | 16 | 21.5 | 42 |
| **i = 9** | 1918 | 155.2 | 80.9 | 18.5 | 23 | 51 |
| **i = 10** | 1632 | 120.2 | 73.7 | 14 | 20 | 36 |
| **average** | ***N*** | ***M* (g)** | ***m* (mg)** | ***D* (cm)** | ***s* (cm)** | ***U*** |
|  | 2036.6 | 163.7 | 79.8 | 18.9 | 24.8 | 51.7 |
| **standard**  **deviation** | **Δ*N*** | **Δ*M* (g)** | **Δ*m* (mg)** | **Δ*D* (cm)** | **Δ*s* (cm)** | **Δ*U*** |
|  | 401.4 | 21.2 | 12.4 | 2.6 | 3.3 | 8.2 |

**Supplementary Table S3**: Number *N* and total mass *M* of all kernel-filled seeds, furthermore diameter *D* and arc length *s* along the diameter in 10 sunflower heads (i = 1, 2, ..., 10) of type nS = naturally South-facing. *U* is the number of unfilled seeds (without kernel).

**sunflower head type nS = naturally South-facing**

| **head** | ***N*_i_** | ***M*_i_ (g)** | ***m*_i_ = *M*_i_/*N*_i_ (mg)** | ***D*_i_ (cm)** | ***s*_i_ (cm)** | ***U*_i_** |
| --- | --- | --- | --- | --- | --- | --- |
| **i = 1** | 1340 | 46.2 | 34.5 | 9 | 10.5 | 22 |
| **i = 2** | 980 | 44.2 | 45.1 | 11.5 | 13 | 30 |
| **i = 3** | 968 | 30.2 | 31.2 | 9.5 | 10.5 | 21 |
| **i = 4** | 981 | 45.2 | 46.1 | 10 | 12.5 | 23 |
| **i = 5** | 985 | 45.2 | 45.9 | 11.5 | 13 | 29 |
| **i = 6** | 894 | 42.2 | 47.2 | 11 | 12 | 28 |
| **i = 7** | 547 | 20.2 | 36.9 | 8.5 | 10.5 | 21 |
| **i = 8** | 1026 | 49.2 | 48.0 | 10.5 | 12.5 | 26 |
| **i = 9** | 844 | 55.2 | 65.4 | 13 | 15 | 33 |
| **i = 10** | 482 | 20.2 | 41.9 | 9.5 | 10 | 21 |
| **average** | ***N*** | ***M* (g)** | ***m* (mg)** | ***D* (cm)** | ***s* (cm)** | ***U*** |
|  | 904.7 | 39.8 | 44.2 | 10.4 | 11.95 | 25.4 |
| **standard**  **deviation** | **Δ*N*** | **Δ*M* (g)** | **Δ*m* (mg)** | **Δ*D* (cm)** | **Δ*s* (cm)** | **Δ*U*** |
|  | 244.2 | 12.1 | 9.4 | 1.4 | 1.6 | 4.4 |

**Supplementary Table S4**: Number *N* and total mass *M* of all kernel-filled seeds, furthermore diameter *D* and arc length *s* along the diameter in 10 sunflower heads (i = 1, 2, ..., 10) of type nZ = naturally Zenit-facing. *U* is the number of unfilled seeds (without kernel).

**sunflower head type nZ = naturally Zenit-facing**

| **head** | ***N*_i_** | ***M*_i_ (g)** | ***m*_i_ = *M*_i_/*N*_i_ (mg)** | ***D*_i_ (cm)** | ***s*_i_ (cm)** | ***U*_i_** |
| --- | --- | --- | --- | --- | --- | --- |
| **i = 1** | 319 | 2.2 | 6.9 | 9 | 9.5 | 14 |
| **i = 2** | 287 | 3.2 | 11.1 | 10.5 | 14 | 18 |
| **i = 3** | 622 | 7.2 | 11.6 | 12 | 17 | 21 |
| **i = 4** | 155 | 1.2 | 7.7 | 8.5 | 11 | 11 |
| **i = 5** | 155 | 2.2 | 14.2 | 8 | 8.5 | 12 |
| **i = 6** | 283 | 6.2 | 21.9 | 9.5 | 10.5 | 13 |
| **i = 7** | 108 | 1.2 | 11.1 | 9 | 11.5 | 10 |
| **i = 8** | 319 | 4.2 | 13.2 | 10 | 11 | 16 |
| **i = 9** | 247 | 2.2 | 8.9 | 9 | 9 | 12 |
| **i = 10** | 411 | 5.2 | 12.7 | 10.5 | 13.5 | 19 |
| **average** | ***N*** | ***M* (g)** | ***m* (mg)** | ***D* (cm)** | ***s* (cm)** | ***U*** |
|  | 290.6 | 3.5 | 11.9 | 9.6 | 11.55 | 14.6 |
| **standard**  **deviation** | **Δ*N*** | **Δ*M* (g)** | **Δ*m* (mg)** | **Δ*D* (cm)** | **Δ*s* (cm)** | **Δ*U*** |
|  | 148.3 | 2.1 | 4.2 | 1.2 | 2.6 | 3.7 |

**Supplementary Table S5**: Number *N* and total mass *M* of all kernel-filled seeds, furthermore diameter *D* and arc length *s* along the diameter in 10 sunflower heads (i = 1, 2, ..., 10) of type tW = tube-wearing, artificially West-facing. *U* is the number of unfilled seeds (without kernel).

**sunflower head type tW = tube-wearing, artificially West-facing**

| **head** | ***N*_i_** | ***M*_i_ (g)** | ***m*_i_ = *M*_i_/*N*_i_ (mg)** | ***D*_i_ (cm)** | ***s*_i_ (cm)** | ***U*_i_** |
| --- | --- | --- | --- | --- | --- | --- |
| **i = 1** | 1206 | 62.2 | 51.6 | 13.5 | 17 | 36 |
| **i = 2** | 983 | 48.2 | 49.0 | 11.5 | 14 | 31 |
| **i = 3** | 1128 | 57.2 | 50.7 | 12.5 | 14.5 | 30 |
| **i = 4** | 1142 | 65.2 | 57.1 | 13.5 | 15.5 | 33 |
| **i = 5** | 1220 | 53.2 | 43.6 | 10.5 | 13 | 25 |
| **i = 6** | 1166 | 67.2 | 57.6 | 12.5 | 15.5 | 31 |
| **i = 7** | 984 | 43.2 | 43.9 | 11 | 13 | 28 |
| **i = 8** | 1210 | 62.2 | 51.4 | 12.5 | 14.5 | 30 |
| **i = 9** | 853 | 31.2 | 36.6 | 9 | 11.5 | 21 |
| **i = 10** | 1135 | 32.2 | 28.4 | 10 | 12.5 | 28 |
| **average** | ***N*** | ***M* (g)** | ***m* (mg)** | ***D* (cm)** | ***s* (cm)** | ***U*** |
|  | 1102.7 | 52.2 | 47.0 | 11.65 | 14.1 | 29.3 |
| **standard**  **deviation** | **Δ*N*** | **Δ*M* (g)** | **Δ*m* (mg)** | **Δ*D* (cm)** | **Δ*s* (cm)** | **Δ*U*** |
|  | 122.0 | 13.2 | 9.1 | 1.5 | 1.6 | 4.2 |

**Supplementary Statistical Table Group T1**: One-way ANOVA and Tukey-Kramer post-hoc tests performed on the average head diameter *D* and diameter’s arc length *s* in sunflower heads of different types defined in Table 1. If critical value < absolute mean difference, then the statistical difference between two head types is significant.

One-way ANOVA for *D*

| **SUMMARY** |  |  |  |  |  |  |
| --- | --- | --- | --- | --- | --- | --- |
| **Groups** | **Count** | **Sum** | **Average** | **Variance** |  |  |
| **tW** | 10 | 116.5 | 11.7 | 2.3 |  |  |
| **nN** | 10 | 114 | 11.4 | 1.2 |  |  |
| **nS** | 10 | 104 | 10.4 | 1.9 |  |  |
| **nE** | 10 | 189 | 18.9 | 6.9 |  |  |
| **nU** | 10 | 96 | 9.6 | 1.4 |  |  |
| **ANOVA** |  |  |  |  |  |  |
| **Source of Variation** | **SS** | **df** | **MS** | **F** | **P-value** | **F crit** |
| **Between Groups** | 556.5 | 4 | 139.1 | 51.1 | 0 | 2.6 |
| **Within Groups** | 122.6 | 45 | 2.7 |  |  |  |
| **Total** | 679.1 | 49 |  |  |  |  |

One-way ANOVA for *s*

| **SUMMARY** |  |  |  |  |  |  |
| --- | --- | --- | --- | --- | --- | --- |
| **Groups** | **Count** | **Sum** | **Average** | **Variance** |  |  |
| **tW** | 10 | 141 | 14.1 | 2.7 |  |  |
| **nN** | 10 | 123 | 12.3 | 0.8 |  |  |
| **nS** | 10 | 119.5 | 11.9 | 2.5 |  |  |
| **nE** | 10 | 248 | 24.8 | 11.0 |  |  |
| **nU** | 10 | 115.5 | 11.6 | 6.8 |  |  |
| **ANOVA** |  |  |  |  |  |  |
| **Source of Variation** | **SS** | **df** | **MS** | **F** | **P-value** | **F crit** |
| **Between Groups** | 1253.3 | 4 | 313.3 | 65.9 | 0 | 2.6 |
| **Within Groups** | 214.1 | 45 | 4.8 |  |  |  |
| **Total** | 1467.3 | 49 |  |  |  |  |

Tukey-Kramer post-hoc tests for *D* and *s*

| **Comparison** | **Abs. mean**  **diff.**  **(T-K test**  **for *D*)** | **Crit. value**  **(T-K test**  **for *D*)** | **Significance**  **for *D*** | **Abs. mean**  **diff.**  **(T-K test**  **for *s*)** | **Crit. value**  **(T-K test**  **for *s*)** | **Significance**  **for *s*** |
| --- | --- | --- | --- | --- | --- | --- |
| **tW vs. nN** | 0.3 | 1.7 | not significant | 1.8 | 1.8 | not significant |
| **tW vs. nS** | 1.3 | 1.7 | not significant | 2.2 | 1.8 | significant  ***s* tW > nS** |
| **tW vs. nE** | 7.3 | 1.7 | significant  ***D* tW < nE** | 10.7 | 1.8 | significant  ***s* tW < nE** |
| **tW vs. nU** | 2.1 | 1.7 | significant  ***D* tW > nU** | 2.6 | 1.8 | significant  ***s* tW > nU** |
| **nN vs. nS** | 1.0 | 1.7 | not significant | 0.4 | 1.8 | not significant |
| **nN vs. nE** | 7.5 | 1.7 | significant  ***D* nN < nE** | 12.5 | 1.8 | significant  ***s* nN < nE** |
| **nN vs. nU** | 1.8 | 1.7 | significant  ***D* nN > nU** | 0.8 | 1.8 | not significant |
| **nS vs. nE** | 8.5 | 1.7 | significant  ***D* nS < nE** | 12.9 | 1.8 | significant  ***s* nS < nE** |
| **nS vs. nU** | 0.8 | 1.7 | not significant | 0.4 | 1.8 | not significant |
| **nE vs. nU** | 9.3 | 1.7 | significant  ***D* nU < nE** | 13.3 | 1.8 | significant  ***s* nU < nE** |

**Supplementary Statistical Table Group T2**: One-way ANOVA and Tukey-Kramer post-hoc tests performed on the average seed number *N*, seed mass *m* and total mass *M* = *Nm* of seeds in sunflower heads of different types (see Table 1). If critical value < absolute mean difference, then the statistical difference between two head types is significant.

One-way ANOVA for *N*

| **SUMMARY** |  |  |  |  |  |  |
| --- | --- | --- | --- | --- | --- | --- |
| **Groups** | **Count** | **Sum** | **Average** | **Variance** |  |  |
| **tW** | 10 | 11027 | 1102.7 | 14882.9 |  |  |
| **nN** | 10 | 13739 | 1373.9 | 95339.4 |  |  |
| **nS** | 10 | 9047 | 904.7 | 59618.9 |  |  |
| **nE** | 10 | 20366 | 2036.6 | 161106.7 |  |  |
| **nU** | 10 | 2906 | 290.6 | 22002.7 |  |  |
| **ANOVA** |  |  |  |  |  |  |
| **Source of Variation** | **SS** | **df** | **MS** | **F** | **P-value** | **F crit** |
| **Between Groups** | 16368240.6 | 4 | 4092060.2 | 58.0 | 0 | 2.6 |
| **Within Groups** | 3176555.9 | 45 | 70590.1 |  |  |  |
| **Total** | 19544796.5 | 49 |  |  |  |  |

One-way ANOVA for *m*

| **SUMMARY** |  |  |  |  |  |  |
| --- | --- | --- | --- | --- | --- | --- |
| **Groups** | **Count** | **Sum** | **Average** | **Variance** |  |  |
| **tW** | 10 | 469.9 | 47.0 | 83.3 |  |  |
| **nN** | 10 | 276.9 | 27.7 | 38.3 |  |  |
| **nS** | 10 | 442.1 | 44.2 | 89.1 |  |  |
| **nE** | 10 | 798.2 | 79.8 | 154.1 |  |  |
| **nU** | 10 | 119.3 | 11.9 | 17.8 |  |  |
| **ANOVA** |  |  |  |  |  |  |
| **Source of Variation** | **SS** | **df** | **MS** | **F** | **P-value** | **F crit** |
| **Between Groups** | 25688.7 | 4 | 6422.2 | 83.9 | 0 | 2.6 |
| **Within Groups** | 3443.0 | 45 | 76.5 |  |  |  |
| **Total** | 29131.6 | 49 |  |  |  |  |

One-way ANOVA for *M*

| **SUMMARY** |  |  |  |  |  |  |
| --- | --- | --- | --- | --- | --- | --- |
| **Groups** | **Count** | **Sum** | **Average** | **Variance** |  |  |
| **tW** | 10 | 522000 | 52200 | 173111111.1 |  |  |
| **nN** | 10 | 368000 | 36800 | 39155555.6 |  |  |
| **nS** | 10 | 398000 | 39800 | 145600000 |  |  |
| **nE** | 10 | 1637000 | 163700 | 1927833333 |  |  |
| **nU** | 10 | 35000 | 3500 | 4455555.6 |  |  |
| **ANOVA** |  |  |  |  |  |  |
| **Source of Variation** | **SS** | **df** | **MS** | **F** | **P-value** | **F crit** |
| **Between Groups** | 149498600000 | 4 | 37374650000 | 81.6 | 0 | 2.6 |
| **Within Groups** | 20611400000 | 45 | 458031111.1 |  |  |  |
| **Total** | 170110000000 | 49 |  |  |  |  |

Tukey-Kramer post-hoc tests for *N*

| **Comparison** | **Abs. mean diff.**  **(T-K test for *N*)** | **Crit. value**  **(T-K test for *N*)** | **Significance**  **for *N*** |
| --- | --- | --- | --- |
| **tW vs. nN** | 271.2 | 303.9 | not significant |
| **tW vs. nS** | 198.0 | 303.9 | not significant |
| **tW vs. nE** | 933.9 | 303.9 | significant  ***N* tW < nE** |
| **tW vs. nU** | 812.1 | 303.9 | significant  ***N* tW > nU** |
| **nN vs. nS** | 469.2 | 303.9 | significant  ***N* nN > nS** |
| **nN vs. nE** | 662.7 | 303.9 | significant  ***N* nN < nE** |
| **nN vs. nU** | 1083.3 | 303.9 | significant  ***N* nN > nU** |
| **nS vs. nE** | 1131.9 | 303.9 | significant  ***N* nS < nE** |
| **nS vs. nU** | 614.1 | 303.9 | significant  ***N* nS > nU** |
| **nE vs. nU** | 1746.0 | 303.9 | significant  ***N* nE > nU** |

Tukey-Kramer post-hoc tests for *m*

| **Comparison** | **Abs. mean diff.**  **(T-K test for *m*)** | **Crit. value**  **(T-K test for *m*)** | **Significance**  **for *m*** |
| --- | --- | --- | --- |
| **tW vs. nN** | 19.3 | 10.7 | significant  ***m* tW > nN** |
| **tW vs. nS** | 2.8 | 10.7 | not significant |
| **tW vs. nE** | 32.8 | 10.7 | significant  ***m* tW < nE** |
| **tW vs. nU** | 35.1 | 10.7 | significant  ***m* tW > nU** |
| **nN vs. nS** | 16.5 | 10.7 | significant  ***m* nN < nS** |
| **nN vs. nE** | 52.1 | 10.7 | significant  ***m* nN < nE** |
| **nN vs. nU** | 15.8 | 10.7 | significant  ***m* nN > nU** |
| **nS vs. nE** | 35.6 | 10.7 | significant  ***m* nS < nE** |
| **nS vs. nU** | 32.3 | 10.7 | significant  ***m* nS > nU** |
| **nE vs. nU** | 67.9 | 10.7 | significant  ***m* nE > nU** |

Tukey-Kramer post-hoc tests for *M*

| **Comparison** | **Abs. mean diff.**  **(T-K test for *M*)** | **Crit. value**  **(T-K test for *M*)** | **Significance**  **for *M*** |
| --- | --- | --- | --- |
| **tW vs. nN** | 15400 | 13950 | significant  ***M* tW > nN** |
| **tW vs. nS** | 12400 | 13950 | not significant |
| **tW vs. nE** | 111500 | 13950 | significant  ***M* tW < nE** |
| **tW vs. nU** | 48700 | 13950 | significant  ***M* tW > nU** |
| **nN vs. nS** | 3000 | 13950 | not significant |
| **nN vs. nE** | 126900 | 13950 | significant  ***M* nN < nE** |
| **nN vs. nU** | 33300 | 13950 | significant  ***M* nN > nU** |
| **nS vs. nE** | 123900 | 13950 | significant  ***M* nS < nE** |
| **nS vs. nU** | 36300 | 13950 | significant  ***M* nS > nU** |
| **nE vs. nU** | 160200 | 13950 | significant  ***M* nE > nU** |

**Supplementary Statistical Table Group T3**: One-way ANOVA and Tukey-Kramer post-hoc tests performed on the average number *U* of unfilled seeds (without kernel) in sunflower heads of different types (see Table 1). If critical value < absolute mean difference, then the statistical difference between two head types is significant.

One-way ANOVA for *U*

| **SUMMARY** |  |  |  |  |  |  |
| --- | --- | --- | --- | --- | --- | --- |
| **Groups** | **Count** | **Sum** | **Average** | **Variance** |  |  |
| **tW** | 10 | 293 | 29.3 | 17.3 |  |  |
| **nN** | 10 | 276 | 27.6 | 8.9 |  |  |
| **nS** | 10 | 254 | 25.4 | 19.4 |  |  |
| **nE** | 10 | 517 | 51.7 | 67.1 |  |  |
| **nU** | 10 | 146 | 14.6 | 13.8 |  |  |
| **ANOVA** |  |  |  |  |  |  |
| **Source of Variation** | **SS** | **df** | **MS** | **F** | **P-value** | **F crit** |
| **Between Groups** | 7350.7 | 4 | 1837.7 | 72.57780411 | 0 | 2.578739184 |
| **Within Groups** | 1139.4 | 45 | 25.3 |  |  |  |
| **Total** | 8490.1 | 49 |  |  |  |  |

Tukey-Kramer post-hoc tests for *U*

| **Comparison** | **Abs. mean diff.**  **(T-K test for *U*)** | **Crit. value**  **(T-K test for *U*)** | **Significance**  **for *U*** |
| --- | --- | --- | --- |
| **tW vs. nN** | 1.7 | 6.43 | not significant |
| **tW vs. nS** | 3.9 | 6.43 | not significant |
| **tW vs. nE** | 22.4 | 6.43 | significant  ***U* tW < nE** |
| **tW vs. nU** | 14.7 | 6.43 | significant  ***U* nU < tW** |
| **nN vs. nS** | 2.2 | 6.43 | not significant |
| **nN vs. nE** | 24.1 | 6.43 | significant  ***U* nN < nE** |
| **nN vs. nU** | 13.0 | 6.43 | significant  ***U* nU < nN** |
| **nS vs. nE** | 26.3 | 6.43 | significant  ***U* nS < nE** |
| **nS vs. nU** | 10.8 | 6.43 | significant  ***U* nU < nS** |
| **nE vs. nU** | 37.1 | 6.43 | significant  ***U* nU < nE** |
